# Current Status and Future Perspectives about Molecular Biomarkers of Nasopharyngeal Carcinoma

**DOI:** 10.3390/cancers13143490

**Published:** 2021-07-12

**Authors:** Pui Yan Siak, Alan Soo-Beng Khoo, Chee Onn Leong, Boon-Peng Hoh, Shiau-Chuen Cheah

**Affiliations:** 1 Faculty of Medicine and Health Sciences, UCSI University, Kuala Lumpur 56000, Malaysia; siakpy@ucsiuniversity.edu.my (P.Y.S.); hohbp@ucsiuniversity.edu.my (B.-P.H.); 2 Cancer Research Centre, Molecular Pathology Unit, Institute for Medical Research, National Institutes of Health, Ministry of Health Malaysia, Shah Alam 40170, Selangor, Malaysia; alk2003@alumni.weill.cornell.edu; 3 Institute for Research, Development and Innovation, International Medical University, Kuala Lumpur 57000, Malaysia; cheeonn_leong@imu.edu.my; 4 School of Postgraduate Studies, International Medical University, Kuala Lumpur 57000, Malaysia; 5 School of Medicine, Taylor’s University, Subang Jaya 47500, Selangor, Malaysia; 6 Centre of Cancer and Stem Cell Research, International Medical University, Kuala Lumpur 57000, Malaysia

**Keywords:** nasopharyngeal carcinoma (NPC), Epstein–Barr virus, epigenetics, biomarkers, therapeutic resistance

## Abstract

**Simple Summary:**

Nasopharyngeal carcinoma is a serious major public health problem in its endemic countries. Up to 80% of NPC patients with locally advanced disease or distant metastasis at diagnosis were associated with poor prognosis and with median survival less than 4 months. The mortality rate of NPC metastasis is up to 91%. To date, there is no available curative treatment or reliable early diagnosis or prognosis for NPC. Discovery and development of reliable early diagnosis and prognosis biomarkers for nasopharyngeal carcinoma are urgent needed. Hence, we have here listed the potential early diagnosis and prognosis biomarker candidates for nasopharyngeal carcinoma. This review will give an insight to readers on the progress of NPC biomarker discovery to date, as well as future prospective biomarker development and their translation to clinical use.

**Abstract:**

Nasopharyngeal carcinoma (NPC) is an epithelial malignancy that shows a remarkable ethnic and geographical distribution. It is one of the major public health problems in some countries, especially Southern China and Southeast Asia, but rare in most Western countries. Multifactorial interactions such as Epstein–Barr virus infection, individual’s genetic susceptibility, as well as environmental and dietary factors may facilitate the pathogenesis of this malignancy. Late presentation and the complex nature of the disease have led it to become a major cause of mortality. Therefore, an effective, sensitive, and specific molecular biomarker is urgently needed for early disease diagnosis, prognosis, and prediction of metastasis and recurrence after treatment. In this review, we discuss the recent research status of potential biomarker discovery and the problems that need to be explored further for better NPC management. By studying the aberrant pattern of these candidate biomarkers that promote NPC development and progression, we are able to understand the complexity of this malignancy better, hence positing our stands better towards strategies that may provide a way forward to the discovery of more reliable and specific biomarkers for diagnosis and targeted therapeutic development.

## 1. Introduction

**Figure 1:**
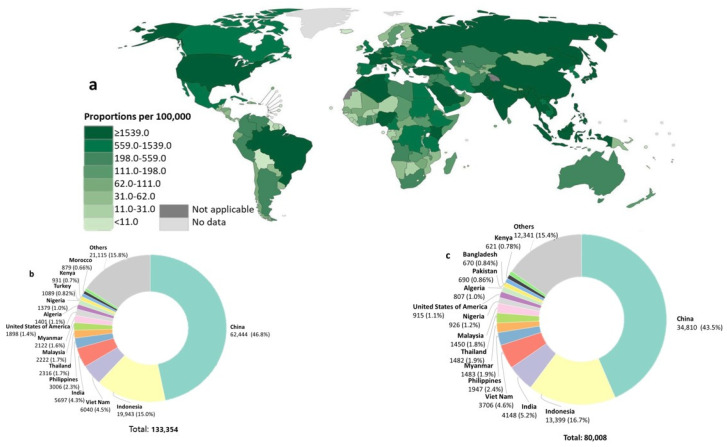
Worldwide distribution of NPC in 2020. (**a**) Map view of estimated number of NPC prevalent cases (5-year) in 2020. Estimated number of (**b**) new cases and (**c**) mortality in 2020. Data from GLOBOCAN 2020.

Nasopharyngeal carcinoma (NPC) is a cancer that arises from the squamous epithelial cells that cover the lateral wall of the nasopharynx [1]. In contrast to head and neck cancers, NPC has a distinct epidemiology, pathology, clinical characteristics, and treatment response [2]. NPC is an endemic form of malignancy in certain parts of the world. It is highly prevalent in parts of North Africa, Alaska, Greenlanders, and Southern Asia especially Southern China, with 50,000 new cases being reported annually (Figure 1) [3]. The top ten countries with the highest number of cases of NPC are China, Indonesia, Vietnam, India, the Philippines, Thailand, Malaysia, Myanmar, the United States of America, and Algeria [4]. Despite its distinct geographical distribution, it is also more likely to occur in certain ethnic groups including Bidayuh, Nagas, and Inuits [5]. Moreover, NPC is more prevalent in men than women (2:1), with an incidence rate of up to 16 per 100,000 men each year [6]. According to GLOBOCAN 2020 [7], 133,354 cases of NPC and 80,008 deaths were reported in 2020 (Figure 1b,c). Owing to its very low prevalence in most developed Western countries, it has often been considered an orphanage disease.

A well-known risk factor of NPC is the Epstein–Barr virus (EBV). Despite that, distinct ethnic and geographical dissemination of NPC indicates both genetic and environmental factors (diet and tobacco smoking) play an important role in its aetiology [2]. Complex interactions of multiple factors including viral infection, an individual’s genetic susceptibility, environmental factors, and dietary factors have driven the pathogenesis of this malignancy.

Up to 80% of NPC patients are diagnosed at advanced stages (clinical stages III and IV) and 10% at distant metastasis, which is associated with unfavourable outcome and poor prognosis [8,9,10,11]. This is mainly due to the fact that it is asymptomatic in its early stages, its high metastatic rate, and its inaccessibility for examination, whereby examination of the local primary tumour in the small curved structure of the nasal cavity is difficult [12]. The common symptoms of NPC include epistaxis, nasal obstruction, hearing loss, otitis media, headache, diplopia, numbness, and neck lump [13,14].

In recent decades, the advancement of diagnostic imaging and the use of concurrent radio and systemic therapy have improved overall prognosis and treatment outcomes [8]. The tumour-node-metastasis (TNM) staging system developed by the American Joint Committee on Cancer and the National Comprehensive Cancer Network (NCCN) is used in treatment decisions for NPC patients at different stages. Radiotherapy (RT) is used as a standard treatment for early stage NPC, while concurrent chemotherapy (CT) followed by adjuvant chemotherapy is the preferred treatment for stages III and IV NPC.

Although overall survival (OS) has improved due to these advanced treatments, there are still many controversies regarding these treatment approaches. For example: (1) patients still encounter tumour recurrence or develop distant metastasis after undergoing RT, especially those in the advanced stages, resulting in death [2]; (2) most patients, especially those in the advanced stages of NPC, did not benefit from the abovementioned NPC treatments [15,16]; (3) a weak tolerance to the high toxic side effects of these therapeutics has led to a delay in treatment, and ultimately death (for example, nasopharynx haemorrhage, a dangerous and serious condition resulting from radiotherapy has led to 35.7% to 100% mortality [13,17]); (4) these treatments eventually allow for tumour progression and emergency due to radio- or chemo-resistance [18,19]; (5) the advanced stages of NPC are associated with poor prognosis and poor response towards the available treatments; and (6) the absence of a reliable prediction tool for NPC recurrence and metastasis. Treatment failure for advanced stages (distant metastasis) is the primary cause of mortality from NPC, accounting for 50,000 deaths annually [10]. Since the 10-year OS rate for stage I patients is as high as 98%, it seems that the mortality rate can be reduced if the NPC is diagnosed at an earlier stage [20]. Currently, the TNM staging system does not provide information on predicting or identifying the risk of NPC progression. This has highlighted the issues of NPC diagnosis and prognosis, as well as treatment. Hence, most studies now focus on uncovering the molecular biomarkers in NPC to improve the early diagnosis approaches and discover prognostic indicators. In the current review, we have reviewed the research status of biomarkers in NPC for early diagnosis and prognosis (metastasis and recurrence).

## 2. Diagnostic and Prognostic Biomarker Discovery for NPC

The use of biomarkers in cancer management has recently been increased with advancements in genomics, proteomics, and transcriptomics, as well as associated technologies. Studying the biomarkers involved in NPC progression and metastasis enables us to understand the disease, identify an individual’s susceptibility to the disease, and predict or monitor patients’ response toward a therapeutic treatment. Based on their role in disease management, biomarkers can be categorised into two groups: (1) prognostics, which allow for the assessment of the risk of clinical outcomes including recurrence, metastasis, and progression; and (2) diagnostic markers, which identify whether an individual has the specific disease or condition.

Therefore, biomarkers can improve the early diagnosis and prognosis approaches by assisting in identifying patients who are susceptible to developing NPC or who are at a high risk or distant metastasis or recurrence. Biomarkers are the key to preventing NPC progression, recurrence, and metastasis, as well as to developing effective therapeutic treatments. With the aid of high throughput ‘omics’ technologies, knowledge on the aetiology, tumorigenesis, and progression of NPC has progressed much faster, thus allowing researchers to identify potential molecular biomarkers. Several types of potential NPC molecular biomarker, including DNA (genomic), mRNA (transcriptomic), protein (proteomic), and metabolite (metabolomics) biomarkers, have been identified (Table 1).

## 3. NPC Diagnostic Biomarkers

### 3.1. NPC Associated Genomic Biomarkers

The discovery of new pathogenic, susceptible genes or mechanisms to assess the risk of NPC has become one of the goals of genomic studies. This is due to NPC being highly complex and multifactorial and characterised by chromosomal aberrations, low mutation occurrence, epigenetic alteration, and the harbouring of cancer specific single nucleotide polymorphisms (SNPs) [1]. Genomic studies have focused on these factors to identify potential biomarkers (Table 1).

(a) NPC associated polymorphism and HLA

With high throughput technologies, including whole-exome sequencing, whole-genome sequencing (WGS), and genome-wide association studies (GWAS), many SNPs associated with NPC risk or pathogenesis have been investigated. For example, polymorphism allele rs5275 in *cyclooxygenase-2* (*COX-2*), rs1024611 in *monocyte chemoattractant protein-1* (*MCP-1*), and rs3216733 in *78-kDa glucose-regulated protein* (*GRP78*) gene promoter are associated with NPC susceptibility [21,22,23]. *MCP-1* is a member of the chemokine family and acts as a potent chemoattractant for immune cells including memory T and lymphocytes. Its association with NPC development via three distinct mechanisms has been proven. These mechanisms are: (i) facilitating tumour cell growth; (ii) modulating the tumour microenvironment (TME) by recruiting several immune cells; (iii) suppressing cytotoxic T-lymphocyte (CTLs) activities [22]. *GRP78* encodes the HSPA78 protein (heat-shock 70-kDa protein 5), which is important for protein folding, where it promotes angiogenesis by upregulating vascular endothelial growth factor (VEGF) and facilitates tumour survival and proliferation [23]. Significant levels of COX-2 were found in 75.58% of NPC patients when compared to people with healthy nasopharynxes. It promotes NPC development by modulating the interaction between tumour cells and myeloid-derived suppressor cells [21]. However, in one contrasting study, this SNP displayed a protective effect on NPC development [62]. The environmental factors such as dietary pattern and geographic areas may explain the contrasting role of this SNP in NPC susceptibility. Moreover, through genetic analysis, the *dendritic cells specific intercellular adhesion molecule 3-grabbing nonintegrin* (*DC-SIGN*) gene variants with SNPs rs7252229, rs735240, and rs4804803 were also found to be associated with NPC susceptibility [24,25]. Polymorphism in *DC-SIGN* with genotype GG at rs735240 and AA at rs2287886 enable dendritic cells to be easily infected by cytomegaloviruses, which have similar a structure to EBV. The glycoprotein on the cytomegaloviruses is able to bind to DC-SIGN and allow the virus to enter the B cells and epithelial cells. In contrast, *DC-SIGN* with AA at rs735240 have reduced *DC-SIGN* expression, which prevents the EBV from infecting DCs and epithelial cells, thus decreasing the NPC risk.

A highly promising study suggested that NPC patients were observed with a high frequency of polymorphic markers in *HLA* genes deposited in chromosome 6p21, which encode MHC [63]. Southern Chinese and others Asian individuals with *HLA-A2-B-46* and *-B17* have a 2–3 times higher chance of NPC development [26]. Chinese people in the US with *HLA-A2* were associated with high NPC susceptibility. Besides that, Caucasians with *HLA-B5* were also associated with NPC susceptibility [27]. Interestingly, Chinese and Tunisians with *HLA-B13*, Caucasians with *HLA-A2*, and all races with *HLA-A11* have 1/3 or 1/2 lower NPC risk [64]. In fact, EBV associated inhibition of HLA expression in NPC, *HLA* gene polymorphism, and EBV have been consistently cited as etiological cofactors that contribute to NPC susceptibility in various areas with varying levels of risk [50]. Low epitope binding efficiency of HLA results in the abrogation of cytotoxic T cell recognition, thus allowing EBV-infected cells to escape from immune surveillance. For example, *HLA-A2*-restricted loss of variant epitope of LMP1 allows for the immune recognition of infected cells in EBV-positive NPC high incidence areas (Southern China and Taiwan) [50]. Although the association between HLA and EBV in NPC is remains controversial, HLA undoubtedly plays a critical role in NPC predisposition as a modulator of immune response against EBV. Further investigation into HLA restricted epitopes and EBV proteins is encouraging.

(b) Chromosome aberration

Genomic studies on aberrant NPC chromosomes have found numerous susceptible genomic events. The chromosomal loss of 3p and 9p was histologically detected in the normal nasopharynx epithelial lining of people in endemic areas, and this seems to be involved in the early stages of NPC transformation [29], while copy number gains in chromosome 12 were found to be one of the signature hallmarks in the early stages of NPC pathogenesis [30]. Previous studies have also revealed that loss of chromosomes in the 1p, 3p, 9p, 9q, 11q, 14q, and 16q regions and frequent copy number gains in chromosomes 1q, 2q, 3q, 4q, 6q, 7q, 8p, 8q, 11q, 12p, 12q, and 17q are very common in the early stages of NPC [1]. Among these chromosome abnormalities, loss of 3p is the most frequent early event in NPC development. NPC patients with 3p loss were at higher risk of death compared to those without 3p loss [30]. These chromosome abnormalities are heritable and can be easily detected, thus screening of chromosome abnormalities could potentially assist in identifying high risk populations.

(c) Copy number alterations

Copy number alteration (CNAs) events in EBV-positive and -negative NPC have also been investigated, and it has been found that CNAs are more frequent in EBV-negative NPC compared to EBV-positive NPC [65]. This suggests that EBV plays an important role in promoting CNAs. Allele loss in 3p21.3 and 9p21 impair several TSGs such as *RASSF1A* and *CDKN2A*. The homozygous deletion of *CDKN2A* (*p16*) and *CDKN2B* (*p15*) located in the 9p21.3 region was observed in primary NPC tumours [31]. Homozygous deletion caused *CDKN2A* inactivation and led to G1/S cell cycle deregulation, eventually supporting EBV in promoting NPC pathogenesis [31].

(d) Signalling pathways

Detection of somatic mutations or aberrant gene regulation in pathways that are involved in NPC is another genomic NPC research area in which we may discover biomarkers. Overexpression of the *epidermal growth factor receptor* (*EGFR*) gene was observed to cause abnormal cell proliferation by activating the downstream cascades RAS/ERK in NPC [32]. Individuals with a higher risk of NPC were observed to have more intense EGFR immunostains, thereby suggesting that *EGFR* is also a promising target for NPC risk prediction [33]. Upon EGFR signalling, pyruvate kinase M2 (PKM2) was activated and it stimulated the gene expression and cancer cell growth by binding to FOSL1 or ANTXR2 promoter [33]. Silencing of EGFR and PKM2 downstream genes such as *FOSL1* or *ANTXR2* could block the EGFR signalling [33]. These reports collectively support that the inhibition of EGFR is important in repressing NPC migration and invasion. Furthermore, correlation of EGFR and mesenchymal-epithelial transition factor (c-MET) pathways has been reported, in which c-MET was activated upon EGFR signalling [66,67]. Aberrantly, activation of MET has triggered metastasis, whereby this aberration was found in up to 72% NPC patients. Although EGFR and c-MET seem like promising biomarkers, they are also commonly upregulated in other cancer metastases.

(e) Viral (EBV and HPV)

In addition to heredity and susceptible human genes, a carcinogenic virus, EBV, was detected in 100% of undifferentiated non-keratinizing NPC patient (type II and III) and up to 95% of NPC cases were EBV-positive in its endemic area [8,68,69,70]. The mechanism of EBV infection in NPC development through carcinogenesis, escape immunosurvilence, epigenetic alteration, and enabling the survival of the tumour has been clearly reviewed in a past study [71]. Therefore, EBV associated biomarkers can be used for diagnosis of early asymptomatic NPC and screening of high risk individuals. NPC patients have been identified through the detection of the EBV DNA fragment BamH1-W found in plasma, and the sensitivity ranges from 69% to 99% [34]. Consistent with these findings, a larger cohort study using plasma EBV-DNA for early NPC diagnosis was reported with a specificity of 97.1% and sensitivity of 98.6% [35]. However, there is also a study that reported that EBV DNA circulating tests showed lower sensitivity in the early stages of NPC [72]. The inconsistency of results among the different studies is probably due to the variations in EBV content and pathological condition of the subject. Therefore, the low reproducibility of this test among the different studies has limited its clinical accuracy in NPC diagnosis. Nevertheless, recent studies have strengthened this biomarker with specific genotype, *A73* gene with A157154C polymorphism and RPMS1 with G155391A polymorphism of EBV in susceptibility to NPC development [20,36].

More recent studies on EBV mediated NPC tumorigenesis has revealed that certain genomic variations in the EBV virus are highly associated with NPC development [37]. Xu et al. identified two distinct EBV variants (162476_C and 163364_T) within the *BALF2* gene from Southern China EBV isolates that contributed up to 83% of the overall risk of developing NPC [37]. Therefore, early NPC can be diagnosed by conducting routine clinical monitoring of high risk NPC populations who have a high risk EBV variant. However, further validation of diagnostic efficacy and functional studies on high risk EBV variants in promoting NPC tumorigenesis are needed.

Although these biomarkers look promising, there are still no susceptibility genes that have gained approval for early diagnosis or prognosis to date. Firstly, due to the genomic status it is hard to match with the respective clinical disease phenotype, thus repetitive verification studies in large cohorts with different omics approaches are required [73]. Secondly, most of the SNPs investigated via GWAS are minor alleles that lack downstream function verification, introducing difficulties for further study in large cohorts [74]. Thirdly, heterogeneity of racial, geographic, and pathological characteristics are a cause of the irreproducibility of studies or variation in research results [75]. Lastly, there are a lack of studies that further discover the interaction of multiple genes instead of single genes in this complex tumour [74]. Hence, multiple genomic biomarkers may require the accurate prediction of NPC susceptibility, metastasis risk, and recurrence possibility. Although the interaction among multiple genes makes the complex mechanism of NPC difficult to understand, advanced technology and mature research design are still pushing the research ahead.

Despite EBV playing an important aetiological role in type II and III NPC, human papillomavirus (HPV) is also suggested as a critical virus in EBV-negative NPC aetiology, especially type I EBV-negative NPC in non-endemic areas such as Finland and America [76]. A study conducted among NPC patients in Finland found that around 62% of NPC patients were EBV-positive, 12% of NPC patients were HPV-positive, and 24% of NPC patients were negative for both [76]. The HPV prevalence in Asia, Europe, and America are 26%, 19%, and 24%, respectively, whereas the HPV prevalence in China is 19%, which is lower than regions outside of China with an HPV prevalence of 23% [77]. Therefore, the prevalence of HPV-positive NPC in endemic areas is lower when compared to non-endemic areas [78,79]. Furthermore, Stenmark et al. (2014) identified a unique subset of Epstein–Barr virus (EBV)-negative nasopharyngeal carcinoma among Caucasian patients that is strongly associated with oncogenic HPV [79]. Hence, this suggests that HPV is a strong surrogate biomarker for HPV-positive NPC [80]. In addition, HPV-positive NPC has distinct clinical features compared to EBV-positive NPC, such as having a better survival rate after radiotherapy [78]. However, HPV-positive NPC has been found to come with an increased risk of locoregional reoccurrence and mortality [79].

Moreover, several studies have demonstrated that HPV and EBV infection are exclusively existent [78,81,82,83]. Intriguingly, there are also studies reporting co-infection of EBV and HPV with NPC from endemic areas [84,85,86]. Therefore, the association between HPV or EBV and NPC remains to be discovered. Furthermore, another study reported no evidence of HPV associated with NPC [87]. This inconsistency is likely due to its relatively low case number, as well as ethnic and geographic differences. Besides that, knowledge about HPV in NPC development is still limited. Further studies are required to confirm these claims. Nevertheless, there are a significant number of NPC cases that are not associated with either EBV nor HPV infection. Hence, screening for EBV and HPV may be insufficient to accurately diagnose NPC.

### 3.2. NPC Associated mRNA Biomarkers

In recent years, miRNA studies have become a focal point in the transcriptomic field as miRNA has been known to play an important role in the cell proliferation, migration, metabolism, invasion, metastasis, and immune escape of NPC [6]. Besides that, the stable expression of miRNA in peripheral circulation allows it to be a reliable marker for early diagnosis. Aberrant expression of miRNA in NPC is related with NPC pathogenesis by abnormal regulation of multiple genetic pathways, thus affecting the cell cycles [88]. Cell phenotypes and functional transcriptomic studies of variation in gene expression may assist in revealing the oncogenic gene and its mechanism in certain pathological states. Therefore, an evaluation and understanding of the miRNA expression profile in NPC might help us to discover a reliable biomarker for diagnosis of early NPC as well as screening high risk populations.

Numerous miRNAs with good diagnostic value have been discovered by using advanced technology, including polymerase chain reaction (PCR) and microarray technology (Table 1). A study revealed that miRNA profiling in between NPC and adjacent nasopharyngeal tissues possessed a different expression pattern [38]. Notably, the oncogenic miR17-92 and miR-155 were upregulated, whereas tumour suppressor miR-34, miR-143, and miR-145 were downregulated [38]. Oncogenic aberrant expression of miR-378, miR-141, miR-144, and miR-205 in NPC have been found to promote NPC pathogenesis by affecting tumour suppression, cell cycle (phosphatase and tensin homolog (PTEN)), and enhanced cell proliferation, invasion, and migration [40,41,42,89].

Consistent findings have revealed that NPC diagnostic accuracy could be enhanced by using a panel of miRNA biomarkers. Liu et al. (2013) reported the sensitivity and specificity of an NPC diagnostic method using five plasma mi-RNAs (miR-16, miR-21, miR-24, miR-155, and miR-378) were 87.7% and 82.0%, respectively [39]. Another study compiling 12-miRNA signatures for early diagnosis of NPC demonstrated an accuracy of up to 100% [90]. These 12-miRNA were found to play an important role in NPC development by modulating its target genes to inhibit NF-κB kinase regulator apoptosis and regulate platelet-derived growth factor receptor α. Collectively, these findings have provided an encouraging message on the use of miRNA as a biomarker for the early diagnosis of NPC.

Recently, tumour-educated platelets that have accurate diagnostic efficiency in various other types of cancer look like a promising avenue for NPC diagnostic marker discovery. Two platelet miRNAs, namely miR-34c-3p and miR-18a-5p, which have been detected in NPC patients and healthy controls, were found to have high diagnostic ability with a sensitivity of 92.59% and specificity of 86.11% [28]. However, further functional and validation studies were not carried out. Nevertheless, it still seems to be promising as the platelets can alter the transcriptome and molecular signal by affecting its pre-mRNA splicing upon instructions given by the tumour [91]. Additionally, in contrast to other samples, its RNA expression is not affected by the genomic DNA, thus the RNA expression truly corresponds to the pathological condition of the cancer.

Furthermore, up to 44 EBV mature miRNAs have been validated to be involved in NPC development and progression [8]. For instance, different polymorphisms in the EBER locus are associated with NPC high risk populations [44]. EBER detection was reported as the most efficient and reliable approach due to its high expression (~1 million copies) in NPC cells and its expression is tumour specific [12]. It has been reported to promote cell proliferation and anti-apoptotic functions and manipulate innate immunity. A GWAS study has identified an EBV variant, designated as HKNPC-EBERvar, having four base deletion SNPs downstream of EBER and highly associated with NPC [44]. Based on this, a genetic risk score can be assigned to each EBV variant that can help to identify high risk populations. Another two EBV miRNAs, miR-BamHI A rightward transcripts (BART)7-3p and miR-BART13-3p, which promote cell proliferation and angiogenesis through the NKIRAS2/NF-κB and AMPK/mTOR/HIF1 signalling pathways, respectively have demonstrated their significant potential for early diagnosis of NPC [8,45,46].

In addition, long non-coding (lnc) RNAs also play an important regulatory role in NPC epigenetics. NPC complex regulatory network is formed by the interaction of lncRNAs, miRNAs, and EBV products [92]. The expression of three lncRNAs, such as metastasis associated with lung adenocarcinoma transcript 1, actin filament-associated protein 1-antisense RNA1, and AL359062, were decreased after treatment, and NPC from healthy controls with an area under the curve (AUC) value of 0.918 was identified [43]. This has evidenced the potential early diagnostic role of these three lncRNAs for NPC.

In view of the progression of current studies in transcriptomics, the use of miRNA or lncRNAs as diagnostic biomarkers for NPC is still a huge gap to explore. This is mainly due to a lack of functional verification and clinical analysis of identified miRNA [93]. High specificity and sensitivity are the most important criteria of diagnostic biomarkers for clinical application. Nevertheless, quantification of miRNA can be a challenging aspect of normalisation and processing, and a comes with a high chance of false negatives or positives [94]. Moreover, it is hard to detect the abundance expression of these biomarkers due to their low molecular weight and the concentration of miRNA in the plasma.

### 3.3. NPC Associated Protein Biomarkers

Proteins are found to be involved in regulating many physiological processes, including immune response, metabolism, and cellular signalling pathways, while tumour cells can utilise the protein by-product to make their favourite proteins, thus affecting anabolism and catabolism, eventually leading to an alteration of protein expression patterns. Therefore, these tumour synthesised oncogenic proteins can be used to reflect the real time state of diseases and used for NPC biomarker research.

Proteomic studies have revealed cancer cell secretomes and found several secreted biomolecules that participate in modulating the TME. Some of these biomolecules are responsible for assisting tumour growth, survival, invasion, and immunosurveillance [95]. Three secreted proteins, namely plasminogen activator inhibitor 1 (PAI-1), fibronectin, and mac-2-binding protein (Mac-2 BP), that are involved in cell migration, differentiation, cell adhesion, morphogenesis, and oncogenic transformation were found to be highly expressed in NPC cells, but either weakly or not expressed in healthy nasopharynx cells [47]. Although they can serve as a potential diagnosis biomarker, their specificity is still uncertain as these proteins were also highly expressed in other cancer types such as lung and breast cancers [47]. Nevertheless, several studies have further identified a panel of secreted protein including cathepsin D, stroma-associated protein periostin, cytokeratin 18, keratin-8, stathmin-1, L-plastin, galectin-1, S100 calcium-binding protein A9 (S100A9, C-C motif chemokine 5) (CCL5), and chloride intracellular channel 1 (CLIC1) were deregulated in NPC only, thereby suggesting these as potential NPC biomarkers for diagnosis [48,49,51,52,53,54,96,97].

Apart from human proteins, oncoproteins encoded by EBV, including EBV latent membrane protein (LMP1, LMP2A and LMP2B) and EBV nuclear antigens (EBNAs) (EBNA1, EBNA2, 3A, 3B, 3C and -LP), are known to take part in NPC development [20,57,58]. The NPC diagnosis sensitivity and specificity of LMP1 are 91.4% and 98%, respectively [55]. A subvariant of EBNA1, P-Thr-sv-5 was identified as a viral marker for undifferentiated NPC screening [56]. Furthermore, the screening of EBV serological markers such as IgA antibodies against early antigen (EA), viral capsid antigen (VCA), and EBNA1 has allowed early diagnosis of NPC as these antibodies are elevated years before NPC is diagnosed [98]. Their sensitivity and specificity were further enhanced by using both EBV-viral capsid antigen (VCA) IgA and EBV-early antigen IgA to screen high risk individuals [99]. For example, examining signature enriched antibodies against early lytic antigens, BALF2 in the two aforementioned high risk EBV variants, as identified in Xu et al., is able to predict the NPC risk [37]. Nevertheless, further diagnostic efficacy studies are necessary. A comparison study on EBV associated NPC biomarker has found that combination of BamHI-W and VCA IgA or EA IgG detection was able to improve the specificity or sensitivity of NPC diagnosis [100]. However, although it looks promising for facilitating NPC diagnosis, the detection rate could vary from 20–100% [101]. This is mainly due to the efficacy varying with different screening tools, thus a standardised tool for serology marker examination is required for further confirmation [98]. Secondly, only ~2% of individuals have elevated VCA/IgA and eventually develop NPC [20]. In addition, the elevation of these antibodies can also be triggered by physical or mental stress [20]. Therefore, these factors mean that these EBV-antibodies have low specificity.

Moreover, there are numerous problems that need to be solved before applying these proteomic biomarkers in clinical diagnostics. Primarily, less advanced high throughput technologies used in past studies have limited the investigation of potential clinical significance biomarkers [102,103]. Secondly, the research design should be able to fully verify and evaluate the biomarker with its cofactors that can affect the result, sensitivity, and specificity of the biomarker [104]. Lastly, similar to the genomic biomarker, there is a lack of further clinical validation of these identified proteins. Nevertheless, the proteomic based biomarker studies have been decreasing recently, probably due to the high cost of the equipment and the difficulty of sample isolation.

### 3.4. NPC Associated Metabolite Biomarkers

Metabolites are a main component that directly execute effector action onto biological processes, which are instructed by upstream genes and proteins [105]. Metabolic disorders are an important feature in cancers because the tumour cells alter the metabolism and retrieve nutrition to sustain cell growth continuously. Therefore, an alteration in the metabolism may reflect the disease phenotypes. Studies into metabolomics have gained increasing attention in recent years for tumour biomarker discovery.

Most of these studies have used high throughput mass spectrometry technology, data processing, system integration, cluster index analysis, and integration with information modelling to look for metabolites that reflect clinical disease phenotypes [106]. Numerous metabolites, including kynurenine, N-acetylglucosaminylamine, N-acetylglucosamine hydroxyphenylpyruvate, pyroglutamate, glucose, and glutamate, have been evaluated as potential biomarkers for early NPC diagnosis [59,60]. Further studies conducted in larger NPC cohorts also validated that a panel of seven metabolites including glycerol 1-hexadecanoate, b-hydroxybutyrate, linoleic acid, arachidonic acid, stearic acid, glucose, and proline provided strong NPC diagnosis from disease free controls, with a sensitivity of 88.0% and a specificity of 92.0% [61].

However, certain metabolites are not NPC specific as glucose and metabolic pathways have often been deregulated in cancer cells due to the oxidative stress and mitochondrial respiration injury. Nevertheless, a panel of metabolites change instead of single metabolites could display the real disease condition. Additionally, NPC metabolomics studies are currently in their infancy stage, hence there is still a lot of potential research value for biomarker discovery.

### 3.5. Cigarette Smoking Associated Biomarkers

Smoking is a confirmed risk factor for NPC development. Many studies have demonstrated cigarette smoking confers a two- to six-fold higher risk of NPC development, with the risk increasing the longer one is a smoker and the more cigarettes one smokes in a day [107,108,109,110,111]. Cigarette smoke is known to contain carcinogenic compounds that cause DNA damage (genome), recruit DNA methyltransferase, hypoxia, activate DNA-binding proteins, and can eventually lead to genome alteration (mutation), disruption of cellular metabolic processes, and epigenetic change [112,113].

TP53 is a commonly mutated TSG in smokers that is associated with an increased risk of cancer development [114]. This cancer related mutation typically occurs in mucosal cells that line the airway, which initiates cancer formation. Intriguingly, compared to other cancer types, NPC was reportedly associated with more epigenetic change (DNA methylation) than mutations [71,115]. The epigenetic effect of cigarette smoking has an impact on TSGs and oncogenes through DNA methylation. For example, hypermethylation in *RASSF1A* and *CDKN2A* is one of the epigenetic changes under NPC development [113]. A study reported that *RASSF1A* and *CDKN2A* were upregulated among smokers compared to non-smokers [116]. This has shed a light on the discovery of smoking associated biomarkers for NPC susceptibility. Nevertheless, there are studies that show no association between DNA methylation in *RASSF1A* and *CDKN2A* and smoking in NPC patients. Therefore, further assessment using other TSGs or oncogene pathways is required to determine the correlation between smoking and epigenetic change in NPC.

Numerous endemic studies have demonstrated no association between smoking and risk of NPC [107,117,118]. However, smoking has been reported to assist in EBV activation [119]. Anti-EBV IgA antibodies (VCA and EBNA1) were found to be higher in smokers with increased NPC risk compared with non-smokers [119]. This has led to the possible role of smoking in increasing NPC risk by altering the host response to EBV infection. Nicotine, a hazardous component in cigarette smoke, is known to have an impact on the immune system, and its long term exposure leads to defection in T-cell proliferation and suppression of antibody production [120]. Therefore, elevation of anti-EBV IgA antibodies among the healthy population (especially smokers) could be a sign of NPC development risk. Nonetheless, the correlation between smoking and EBV infection is still indecisive. There is also a possibility that smoking-associated NPC carcinogenesis may work through other mechanisms. Noteworthy, there are other environmental factors, such as diet, lifestyle, and drug use, that may also contribute to DNA methylation or affect the host immune system. Therefore, it is imperative to conduct a more comprehensive study by incorporating the other environmental factors to validate the aforementioned findings.

## 4. NPC Prognosis Biomarkers

Up to 40% of NPC patients have disease recurrence or distant metastasis even after they receive a series of CT or RT [121]. This indicates that tumour cells are able to recover from damaged cells and survive by having resistance to current therapies (CT or RT). Therefore, prediction of NPC recurrence or metastasis risk after treatment is crucial since it is the major cause of mortality in NPC patients. Particularly, molecular components that are metastasis susceptible or capable of affecting the radio- or chemo-sensitivity can be used as a prognosis biomarker (Table 2).

Several studies have revealed that numerous SNPs in different genes are correlated with poor prognosis toward RT and even the development of dermatitis and oral mucositis after treatment. For example, Yu et al. (2016) reported gene polymorphisms in Wnt/β-catenin including *catenin β-1* gene rs1880481, rs3864004, *glycogen synthase kinase-3β* (*GSK-3β*) gene rs3755557 and *adenomatous polyposis coli* (*APC*) gene rs454886 were associated with poor responses to RT [122]. Other gene polymorphisms, such as *X-ray repair cross-complementing 1* (*XRCC1*) rs25489, *calcitonin receptor* rs2528521, *XRCC1* Codon399, and *valosin-containing protein* rs2074549, which can predict therapeutic outcomes and toxic side effects, were also investigated [123,124,125,126]. Besides that, certain polymorphisms were found to be susceptible to metastasis. For instance, the Chinese population with *IL-13* rs20541 polymorphisms was reported to be susceptible to metastasis [154]. Furthermore, the TT genotype of rs20541 and T-C-T haplotype are significantly associated with a higher risk of metastasis and poor prognosis, whereas CT/CC genotypes are associated with decreased risk of metastasis in NPC. SNPs in *excision repair 1 endonuclease non-catalytic subunit* (*ERCC1*) of C118T genotype was discovered as a strong indicator for excellent prognosis in RT for EBV-negative NPC, thus it can help to avoid excess or overtreatment with CT in these patients [127]. All these SNPs could be useful as a prognostic biomarker for NPC treatment.

One study acknowledged the value of EBV-DNA for early NPC recurrence after treatment [155]. Most of the patients had EBV-DNA elevated prior to the disease recurrence [33]. The accuracy, sensitivity, and specificity of recurrence diagnostic using EBV-DNA were 87.0%, 82.3%, and 80.0%, respectively [33]. In another study, the circulating EBV-DNA concentration was found to be higher in recurrent NPC plasma compared to primary NPC plasma, thus implying that recurrence risk can be predicted by detecting the circulating EBV-DNA [156]. The National Comprehensive Cancer Network also recommends monitoring NPC patients with EBV-DNA [157]. This EBV-DNA biomarker was further strengthened by combination with a predictive tool, namely distant metastasis gene signature (DMGN), which constitutes 13 genes including *DCTN1*, *YBX3*, *GRM4*, *HDLBP, POLR2M*, *CLASP1*, *CBR3*, *FNDC3B*, *WSB2*, *LRIG1*, *ANXA1*, *WNK1*, and *CXCL10* to examine whether the patients can benefit from concurrent CT. The patients with the higher predicted metastasis risk would have less sensitivity to concurrent CT [128].

Moreover, by looking at mRNA involved in NPC progression, the subtype of disease, prognosis, and therapeutic effect in NPC could be predicted [93,158,159]. For example, analysed miRNA expression profile of radioresistant and radiosensitive NPC cell lines by next generation deep sequencing have revealed that downregulation of miR-203, miR-324-3p, miR-93-3p, and miR-4501 and upregulation of miR-371a-5p, miR-34c-5p, and miR-1323 contribute to mediating radio-resistance in NPC [129,130,148]. Additionally, miR-574-5p, miR-9 and miR92a, which modulate the expression of MHC class I and interferon-regulated genes associated with NPC metastasis, could potentially be non-invasive blood-based biomarkers for metastasis prediction [132,133]. RNA sequencing of NPC patients’ peripheral blood mononuclear cells (PBMC) before and after RT has revealed 11 potential mRNA prognostic biomarkers for NPC for post-RT evaluation [160]. RNA_0000285 at homeodomain interacting protein kinase 3 (HIPK3) was observed in high level radio-resistance NPC patients and low radiosensitive NPC patients, thus showing its ability to predict NPC radiosensitivity [136].

Furthermore, as mentioned previously, the residue of cigarette smoke promotes cancer progression. Cigarette smoke was found to be associated with poor prognosis of chemotherapy and radiotherapy. Nicotine in cigarette smoke promoted chemoresistance by affecting the ATP-biding cassette transporter G2 via downregulation of miR-296-3p and Akt-mediated pathways [134,135]. Furthermore, hypoxia induced through smoking can facilitate tumour angiogenesis, invasion, reoccurrence, and metastasis. Therefore, the downregulation of miR-296-3p in patients could be a potential prognosis or predictive biomarker for recurrence and metastasis.

In contrast to DNA and RNA based biomarker studies, proteomics studies are more efficient in their evaluation and prognosis of NPC treatment. The interaction among the proteins in various signalling pathways involved in NPC carcinogenesis can be used to reflect the real-time condition of the disease progression [145,161,162]. Therefore, the clinical outcome of each NPC treatment can be evaluated. Several proteins in the cellular signalling pathway have been reported to contribute resistance towards CT or RT. Initially, simultaneous overexpression of insulin-like growth factor-1 receptor (IGF-1R) and EGFR was found to be involved in chemo- and radio-resistance [137,138]. Other signalling molecules in this pathway, like an activated protein, C-Jun activation domain-binding protein-1 (Jab1) and its downstream protein, namely glutathione S-transferase P1 (GSTP1), were found overexpressed and abnormally methylated, respectively [139,163]. This aberration contributed to radio- and drug-resistance (such as resistance to paclitaxel) [138]. Secondly, Notch signalling, Wnt/β-catenin, and the NF-κB signalling pathway, which is involved in tumorigenesis of many cancer types, has been reported to be relevant to radio-resistance in NPC cells. In the NF-κB signalling pathway, NF-κB p65 and Akt Epithelial cell adhesion molecule (EpCAM) promoted EMT, which initiates metastasis by endowing cancer cells with radio-resistance. Increasing levels of phosphorylated GSK-3*β* in EBV infected individuals results in higher levels of β-catenin [141]. β-catenin can activate multiple downstream growth signalling components, such as cyclin D1 and c-Myc, and interact with interleukin-8 (IL-8), *RASSF1*, E-cadherin, and N-cadherin, thereby leading to NPC carcinogenesis [164]. β-catenin forms a complex with E-cadherin for cell adhesion, and also suppresses metastasis [165]. However, in metastatic NPC, the E-cadherin was highly downregulated and the level of E-cadherin is lowest when compared to non-cancerous and primary NPC cells [140,166]. Correlation of N-cadherin and β-catenin in NPC highlighted that both expressions promoted NPC metastasis and poor prognosis. Therefore, the prognosis or metastasis risk of NPC patients can be predicted by looking at the level of β-catenin and E-cadherin complex. Despite that, Post-translational modifications like protein glycosylation involved in malignant transformations also confer therapeutic resistance in NPC. N-glycosyltransferase-V (GnT-V), an enzyme function in glycosylation, upregulation was associated with promoting cell proliferation, anti-apoptosis functions, and upregulation of *Bcl2* gene expression, thus conferring radio-resistance to NPC [142,143,144].

Additionally, RT resistant patients can be identified by assessing the expression patterns of certain serum proteins that participate in tumorigenesis. For example, secreted protein acidic and high cysteine, serpin family D member 1S, complement C4B, peptidylprolyl isomerase B, and a family with sequence similarity 173 member A can discriminate RT resistance patients with a sensitivity of 94.1% and specificity of 92.6% [145]. In a comparative study on radioresistant and sensitive control NPC cell lines, upregulation of non-metastatic clone 23, isoform H1 (Nm23 H1), maspin, GRP78, and manganese superoxide dismutase (Mn-SOD) and downregulation of 14-3-3 protein sigma (14-3-3σ) and annexin A1/A3 were found to be associated with radio-resistance [131,146,147,149,150]. Another comparative study on proteomic profiles of NPC and healthy nasopharynx cells also revealed that deregulation of stathmin, 14-3-3σ, annexin, and cathepsin D were associated with NPC metastasis and recurrence [167,168]. In addition, differentially expressed serum proteins such as keratin 1, serum amyloid A protein (SAA), and heat shock protein (HSP70), which are involved in chemoresistance (cis-Diamminedichloroplatinum) and radio-resistance in NPC, were reported as a potentially useful biomarker in NPC recurrence diagnosis [151,152,153,169,170].

Nevertheless, none of these candidates have been approved for clinical application to date. This is because most of the studies did not evaluate the sensitivity and specificity of biomarker prognosis. The research design also did not consider the aforementioned cofounding factors such as type of treatment and clinical stages, which could affect the results. For instance, the efficacy of a prognostic biomarker should be evaluated in the dual effects of RT and CT. A standard prognostic biomarker efficacy evaluation and result verification procedure should be developed. This procedure should include the variable factors that can affect the reproducibility of biomarker validation results, which are conducted across different laboratories.

## 5. Future Perspective and Challenges

An ideal biomarker for early detection of NPC should be specific to individuals who are about to develop NPC and stable across time. Besides that, an ideal prognosis biomarker should be able to predict the clinical outcome of a specific treatment given to the patients and predict which NPC patients are about to develop metastasis or disease recurrence after treatment. In addition, the level of biomarker should be able to correlate with the tumour burden in order to reflect the progression and regression. With new biomarker-based diagnostic or prognostic tools, personalised medicine that is tailored to the characteristics of respective individual NPC patients can be developed. Over the long term, patients’ welfare can be improved by preventing the disease progression, thus enhancing the disease management and eventually leading to better health outcomes.

The advancements in genomic, transcriptomic, proteomic, and metabolomic research has allowed us to study the disease and discover biomarkers that facilitate the identification of the diagnosis, prognosis, and characteristics of patients. However, although many NPC biomarkers discovered to date look promising, none of these biomarkers are approved for clinical application. This is mainly due to a lack of reproducible validation of result of identified biomarkers across different laboratories like previously mentioned. In fact, there are several factors that can lead to variation of efficacy evaluation for the same biomarker. Firstly, it is due to the genomic instability feature of the tumour and multiple genetic aberrations needed to trigger NPC development and metastasis. The detection of biomarkers at a particular time might not reflect future genetic alteration due to the underlying genomic instability. Secondly, the complexity of the tumorigenesis process and heterogeneity across individual and tumour microenvironments make it unlikely that a single diagnosis biomarker can be effective. Cancers are made up of numerous cell types. Therefore, there is a complex cross talk of interaction between tumour and surrounding stromal cells (tumour microenvironment) in facilitating the progression of early lesion and metastasis, which are not captured by these biomarkers, while more advanced 3D heterotypic multicellular cells with more aggressive metastasis and therapeutic resistance features than 2D cancer cells will provide better simulation of the in vivo TME for biomarker discovery [171,172]. However, most of the studies used 2D NPC cell line instead of 3D heterotypic multicellular tumour cells. Turning our attention to aberrant alterations in TME could potentially direct us towards discovering reliable biomarkers, as well as targets for therapy or personalised medicine development. Thirdly, it is likely that additional genetic events are involved in NPC progression but have yet be discovered. Besides that, the small sample sizes and lack of replicated studies also limits the progress of developing diagnosis biomarkers.

Moreover, cancer involves synergistic pathogenesis alteration such as change in gene mutation, protein synthesis, transcription, and metabolism. Hence, it is impossible to explore the whole complex network of cell signalling pathways and biological processes of NPC carcinogenesis and progression by just looking at a single omics. Integration of multi-omics is required for tumour marker development. Integration of two or more kinds of omics can allow more comprehensive study of the specific molecules at multiple levels (phenotype and regulatory mechanisms), thereby compensating for the lack of data in any single omics, and therefore enhancing the reliability of the biomarker.

Furthermore, it is also impossible to diagnose and monitor this complex disease with only a single biomarker. Therefore, multiple biomarkers with different omics should be considered. The development of cost-effective multiplex diagnosis assays is required to cross detect several NPC biomarkers in order to achieve a more accurate diagnosis. However, using different types of biomarkers for diagnosis is tedious, expensive, and patients might suffer when numerous biopsies need to be done. Despite that, technology advancements in detection assays or screening approaches are important to propel biomarker development. For example, advancements in molecular sequencing will help in identifying genomic change events, including DNA methylation, structural rearrangement, high risk alleles, miRNA, etc.

In addition, there are several challenges that need to be overcome before the biomarker can be translated to clinical use. Firstly, biomarker based diagnostic or prognostic approaches are less adapted to the healthcare systems of many countries. It is to do with the current reimbursement system and less to do with referring the value of these biomarker based diagnostic or prognostic tools in health care settings. In order to support the integration of biomarkers into healthcare systems, evidentiary standards and procedures need to be developed to assess and manipulate the biomarkers based on diagnostic or prognostic tools. For example, EBV DNA has been known to have potential uses in early NPC diagnosis and prognosis, but its efficacy varies with different testing approaches. Hence, in order to evidence its efficacy in NPC detection, a harmonised validation approach should be developed and conducted across different laboratories [173]. Secondly, similar stringent test designs and randomised clinical trials in biomarker development should also be practiced prior to the approval of a pharmaceutical for clinical use.

Apart from this, socio-economic factors are another set of issues that affect cancer care pathways, especially predictive biomarker tests. Undoubtedly, low socio-economic status remains a barrier for patients to access treatment and cancer care, as well as lack of practice using predictive biomarker tests in health care systems. Cancer Research UK highlighted that most cancer patients received targeted therapy without prior molecular biomarker testing [174]. This may result in poor prognosis or overtreatment. In fact, less access to biomarker testing has overwhelmed the value of biomarkers. Hence, Medicaid and other relevant programs should be expanded in order to increase the awareness, patient’s perspective, experience, and access to biomarker-based technology. Furthermore, the industry should also involve new business models that provide support for the development of molecular diagnostic biomarkers.

In short, future research on NPC biomarkers should have: (1) sufficient efficacy evaluation to provide supportive data for further clinical research; (2) integration of multiple disciplines, including regional, environmental, lifestyle, and dietary factors of NPC patients; (3) integration of multi-omics; (4) a call for an international collaboration and effort to involve larger cohorts and diversified populations to demonstrate its usefulness and reliability; (5) further exploration including novel clinical issues in study design.

## Figures and Tables

**Table 1:** Potential biomarkers for early diagnosis of NPC.

Biomolecules	Full Name	Role	Aberration	Sources
Genomic biomarkers				
*COX-2*	*Cyclooxygenase-2*	Cell proliferation, apoptosis	Polymorphism in rs5275	[21]
*MCP-1*	*Monocyte chemoattractant protein-1*	Monocytes or macrophages migration and infiltration	Polymorphism in rs1024611	[22]
*GRP78*	*Glucose-regulated protein*	Apoptosis	Polymorphism in rs3216733	[23]
*DC-SIGN*	*Dendritic cells specific intercellular adhesion molecule 3-grabbing nonintegrin*	Induced immune cells	Polymorphism in rs7252229, rs735240, rs4804803 or rs2287886	[24,25]
*HLA-A2-B46* (Chinese)	*Human leukocyte antigen-A2-B46*	Immune response	Polymorphism in chromosome 6p21	[26,27]
*HLA-A2-B-17* (Chinese)	*Human leukocyte antigen-A2-B-17*	Immune response		
*HLA-B5* (Caucasians)	*Human leukocyte antigen-B5*	Immune response		
*IL-13*	*Interleukin-13*		Polymorphism in rs20541 (TT genotype)	[28]
Chromosome 3p and 9p	N/A	N/A	Chromosomal loss	[29]
Chromosome 12	N/A	N/A	Gain number	[30]
*RASSF1*	Ras association (RalGDS/AF-6) domain family member 1A	Tumour suppression, cell growth, proliferation	copy number variant in in 3p21	[1]
*CDKN2A*, *CDKN2B*	Cyclin-dependent kinase inhibitor 2A, 2B	Tumour suppression, cell cycle	Allelic deletion in 9p21.3	[31]
EGFR	Epidermal growth factor receptor	Cell proliferation, cell cycles, apoptosis	Upregulation	[32,33]
*BamH1-W*	*Bacillus amyloliquefaciens 1 WZhet*	Viral replicative cycle	Upregulation	[34,35]
*A73*	N/A	Cell proliferation and angiogenesis	Polymorphism in A157154C	[20,36]
*RPMS1*	N/A	Cell proliferation and angiogenesis	Polymorphism in G155391A	
*BALF2*	N/A	Viral infection and replication	EBV variants with 162476_C or 163364_T	[37]
**miRNA biomarkers**				
miR17-92	MicroRNA17-92	Targeting PTEN and apoptosis protein	Upregulation	[38]
miR-155	MicroRNA-155	Leucosis	Upregulation	[39]
miR-378	MicroRNA-378	Affect tumour suppression, cell cycle	Upregulation	[40,41]
miR-141	MicroRNA-141			
miR144-3p	MicroRNA-144-3p	Targeting PTEN/Akt, cell cycle, apoptosis	Upregulation	[42]
miR-17-5p	MicroRNA-17-5p			
miR-20a-5p	MicroRNA-20a-5p			
miR-20b-5p	MicroRNA-20b-5p			
miR-205-5p	MicroRNA-205-5p			
miR-16	MicroRNA-16	Cell proliferation, invasion	Upregulation	[39]
miR-21	MicroRNA-21	Targets PDCD4, PTEN, SPRY, ERCK, and Bcl-2 family proteins		
miR-24	MicroRNA-24	Epithelial-to-mesenchymal transition	Upregulation	
miR-146a		Inflammation	Upregulation	[6]
miR-34	MicroRNA-34	Tumour suppression	Downregulation	[38]
miR-143	MicroRNA-143	Tumour suppression		
miR-145	MicroRNA-145	Tumour suppression		
let-7b-5p	MicroRNA let-7b-5p	Cell proliferation	Downregulation	[42]
miR-140-3p	MicroRNA-140-3p	Cell proliferation		
Platelet miR-34c-3p	MicroRNA-34c-3p	Tumour suppression	Upregulation	[28]
Platelet miR-18a-5p	MicroRNA-18a-5p	Tumour suppression		
MALAT1	metastasis associated with lung adenocarcinoma transcript 1	Invasion	Upregulation	[43]
AFAP1-AS1	actin filament-associated protein 1-antisense RNA1	Invasion		
AL359062	N/A	N/A		
EBER	Epstein–Barr encoding region	Cell proliferation, apoptosis, innate immunity	Four base deletion SNPs	[44]
miR-BART7-3p	BamH1 A rightward transcript 7-3p	Cell proliferation targeting NF-κB signalling, angiogenesis targeting AMPK/mTOR/HIF1 signalling	Upregulation	[8,45,46]
miR-BART13-3p	BamH1 A rightward transcript 13-3p	Cell proliferation targeting NF-κB signalling, angiogenesis targeting AMPK/mTOR/HIF1 signalling		
**Protein biomarkers**				
PAI-1	Plasminogen activator inhibitor 1	Angiogenesis, signalling activities	Upregulation	[47]
Fibronectin	N/A	Cell adhesion		
Mac-2 BP	Mac-2-binding protein	Cell adhesion		
CTSD	Cathepsin D	Apoptosis	Upregulation	[48]
POSTN	Periostin	Cell adhesion	Upregulation	[49]
CK18	Cytokeratin 18	Transcription	Upregulation	[50]
KRT8	Keratin-8	Tumour necrosis factor-mediated signaling pathway, cell differentiation	Upregulation	[48]
STMN1	Stathmin-1	Signal transduction		
LCP1	L-plastin	Cell differentiation	Upregulation	[51]
LGALS1	Galectin-1	Apoptosis	Upregulation	[52]
S100A9	S100 calcium-binding protein A9	Cell proliferation, innate immunity, apoptosis	Upregulation	[51]
CCL5	C-C motif chemokine 5	Cell adhesion, migration, apoptosis	Upregulation	[53]
CLIC1	Chloride intracellular channel 1	Cell cycle, signal transduction	Upregulation	[54]
LMP1	Latent membrane protein	Signalling activities	Upregulation	[55]
P-Thr-sv-5	N/A	Gene expression (sub-variant of EBNA1)	subvariant of EBNA1	[56]
EBNA1/IgA	EBV nuclear antigens immunoglobulin A	Antibody against EBV antigen	Increased level	[57,58]
VCA/IgA	Viral capsid antigen immunoglobulin A	Antibody against EBV antigen		
BALF2/Ab	BALF2 antibodies	Antibody against EBV antigen	Increased level	[37]
**Metabolite biomarkers**				
kynurenine	N/A	Metabolism	Upregulation	[59]
N-acetylglucosaminylamine	N/A	Metabolism		
N-acetylglucosamine hydroxyphenylpyruvate	N/A	Metabolism		
Pyroglutamate	N/A	Metabolism	Upregulation	[60]
Glucose	N/A	Metabolism		
Glutamate	N/A	Metabolism		
Glycerol 1-hexadecanoate	N/A	Metabolism	Upregulation	[61]
b-hydroxybutyrate	N/A	Metabolism		
Arachidonic acid	N/A	Metabolism		
Stearic acid	N/A	Metabolism		
Linoleic acid	N/A	Metabolism		
Proline	N/A	Metabolism		

N/A. Not available.

**Table 2:** Potential prognosis and predictive biomarkers for NPC therapeutic resistance or metastasis and recurrence after treatment.

Biomolecules	Name	Role	Aberration	Sources
*β-catenin 1*	Beta-catenin1	Activate multiple downstream growth signalling components such as cyclin D1 and c-Myc	Polymorphism in rs1880481 or rs3864004	[122]
GSK-3*β*	*glycogen synthase kinase-3β*	Cell growth, metabolism, gene transcription, protein translation, cytoskeletal organisation	Polymorphism in rs3755557	
*APC*	*adenomatous polyposis coli*	Cell adhesion	Polymorphism in rs454886	
XRCC1	X-ray repair cross-complementing 1	DNA repair	Polymorphism in rs25489 or Codon399	[123,124,125,126]
*CT*	*Calcitonin receptor*	Calcium homeostasis	Polymorphism in rs2528521	
*VCP*	*Valosin-containing protein*	Proteolysis	Polymorphism in rs2074549	
*IL-13*	*Interleukin-13*	Chinese population with IL-13 rs20541	Polymorphisms in rs20541	[28]
*ERCC1*	*Excision repair 1 endonuclease non-catalytic subunit*	DNA repair	Polymorphism with C118T genotype	[127]
EBV-DNA	Epstein–Barr virus-DNA	EBV genome	Upregulation	[33]
*YBX3*	Y-Box Binding Protein 3	Apoptosis, Gene expression	Upregulation	[128]
*CBR3*	Carbonyl reductase 3	Xenobiotic metabolic process		
*LRIG1*	Leucine-rich repeats and immunoglobulin-like domains 1	Negative regulator of tyrosine kinases signalling		
*CXCL10*	Chemokine C-X-C motif ligand 10	Chemokine receptors recruit tumour infiltrating T-lymphocytes, tumour microenvironment		
*DCTN1*	Dynactin-1	G2/M transition of mitotic cell cycle	Downregulation	
*GRM4*	Glutamate metabotropic receptor 4	Tumour suppression		
*HDLBP*	High density lipoprotein binding protein	Cholesterol metabolic process		
ANXA1	Annexin	Cell cycle, apoptosis		
*POLR2M*	RNA polymerase II subunit M	Negative regulator of transcriptional		
*CLASP1*	Cytoplasmic linker associated protein 1	Dynamic microtubules stabilization		
*FNDC3B*	Fibronectin type III domain-containing protein 3B	Positive regulator of adipogenesis		
*WSB2*	WD repeat and SOCS box-containing protein 2	Protein ubiquitination, post-translation modification		
*WNK1*	lysine deficient protein kinase 1	T-cell receptor signalling pathway		
miR-203	MicroRNA-203	Targeting IL-8/Akt signalling	Downregulation	[129]
miR-324-3p	MicroRNA-324-3p	Tumour suppression	Downregulation	[130,131]
miR-93-3p	MicroRNA-93-3p	Targeting Wnt/β-catenin signalling		
miR-4501	MicroRNA-4501	Cellular process		
miR-371a-5p	MicroRNA-371a-5p	Cellular pathway, apoptosis	Upregulation	
miR-34c-5p	MicroRNA-34c-5p	Cell proliferation, apoptosis, targeting JAK2/STAT3 signalling pathway		
miR-1323	MicroRNA-1323	DNA repair		
miR-9	MicroRNA-9	MHC class I and interferon-regulated gene expression	Downregulation	[132]
miR-92a	MicroRNA-92a	Invasion, migration	Upregulation	[133]
miR-574-5p	MicroRNA-574-5p	Mesenchymal transition	Downregulation	[9]
miR-296-3p	Micro-296-3p	Cytoplasmic Translocation of c-Myc	Downregulation	[134,135]
RNA_0000285		homeodomain interacting protein kinase 3 (HIPK3)	Upregulation	[136]
*EGFR*	Epidermal growth factor receptor	Cell proliferation, cell cycles, apoptosis	Upregulation	[137]
GSTP1	Glutathione S-transferase P1	Cell adhesion, apoptosis, negative regulator of NF-kB signaling	Methylation	[138]
IGF-1R	Insulin-like growth factor-1 receptor	Cell proliferation, cell cycles and apoptosis	Upregulation	[137]
Jab1	C-Jun activation domain-binding protein-1	Cell proliferation, targeting negative regulator proteins and tumour suppressors (p27 and p53)	Upregulation	[139]
EMT	Epithelial-to-mesenchymal transition	Carcinogenesis and metastatic progression	Upregulation	[140]
β-catenin	N/A	Activate multiple downstream growth signalling components such as cyclin D1 and c-Myc	Upregulation	[141]
E-cadherin	N/A	Cell adhesion, tumour suppression	Downregulation	
*GnT-V*	N-acetylglucosaminyltransferase-V	Protein glycosylation, cell proliferation	Upregulation	[142]
*Bcl2*	B-cell lymphoma 2	Apoptosis	Upregulation	[143,144]
*SPARC*	Secreted protein acidic and Cysteine rich	Extracellular matrix synthesis, cell shape	Upregulation	[145]
*ERPIND1*	Serpin family D member 1S	Invasion		
*C4B*	Complement C4B	Component of the classical activation pathway		
*PPIB*	Ppeptidylprolyl lsomerase B	Cyclosporine A-mediated immunosuppression		
*FAM173A*	Family with sequence similarity 173 member A	Adenine nucleotide translocase		
Maspin	Mammary serine protease inhibitor	Tumour suppression	Upregulation	[146,147]
GRP78	Glucose-regulated protein	Apoptosis		
Mn-SOD	Manganese superoxide dismutase	Apoptosis		
14-3-3σ	14-3-3 protein sigma	Cell cycle arrest, DNA damage response, signal transduction	Downregulation	
ANXA1,3	Annexin A1, A3	Cell cycle, apoptosis	Downregulation	[148,149,150]
Nm23 H1	Non-metastatic clone 23, isoform H1	TGF-β signaling	Upregulation	
KRT1	Keratin 1	Angiogenesis	Upregulation	[151]
SAA	Serum amyloid A	MAPK activities, innate immune response	Downregulation	[152]
HSP27	Heat shock protein 27	Apoptosis, cell differentiation	Upregulation	[153]

N/A. Not available
